# Foundational principles for young intensivists to drive better outcomes: the bedside application of physiology

**DOI:** 10.62675/2965-2774.20260286

**Published:** 2026-01-09

**Authors:** Patricia Rieken Macedo Rocco, Marcelo Park, Jan Bakker

**Affiliations:** 1 Universidade Federal do Rio de Janeiro Instituto de Biofísica Carlos Chagas Filho Rio de Janeiro RJ Brazil Laboratory of Pulmonary Investigation, Instituto de Biofísica Carlos Chagas Filho, Universidade Federal do Rio de Janeiro - Rio de Janeiro (RJ), Brazil.; 2 Universidade de São Paulo Faculdade de Medicina Intensive Care Unit, Emergency Department, Hospital das Clínicas São Paulo SP Brazil Intensive Care Unit, Emergency Department, Hospital das Clínicas, Faculdade de Medicina, Universidade de São Paulo - São Paulo (SP), Brazil.; 3 Erasmus MC University Medical Center Department of Intensive Care Adults Rotterdam Netherlands Department of Intensive Care Adults, Erasmus MC University Medical Center - Rotterdam, Netherlands.

## INTRODUCTION

In the rapidly evolving field of intensive care medicine, young intensivists strike a balance between technological advancements and a strong foundation in physiological reasoning. While advances in imaging, biomarkers,^(1)^ and Artificial Intelligence (AI) have transformed diagnostic and therapeutic strategies, the bedside application of core physiological principles remains essential for optimizing patient outcomes. This perspective highlights the enduring value of physiology in critical care, examines the barriers to its bedside application, and provides practical strategies for integrating physiology-driven care into daily practice.

## WHY BEDSIDE PHYSIOLOGY MATTERS

Critical illness is characterized by rapid shifts in respiratory mechanics, hemodynamics, acid-base balance, and autoregulation - all of which demand real-time clinical interpretation. Mastery of physiology enables clinicians to move beyond protocolized care, tailoring interventions to match each patient's unique pathophysiology.

Personalized ventilation strategies targeting driving pressure or compliance, rather than fixed tidal volumes, have been shown to improve outcomes in acute respiratory distress syndrome.^(2)^ Similarly, fluid management guided by dynamic indices, such as passive leg raising or pulse pressure variation, reduces the risk of fluid overload compared to static measures like central venous pressure.^(3)^ Physiological monitoring is also critical for the safe initiation of mobilization and rehabilitation in critically ill patients.^(4)^ Bedside respiratory mechanics assessments, including esophageal pressure monitoring and pressure-time product calculations, optimize ventilator settings to protect both lungs and diaphragm.^(5)^

Acid-base balance further exemplifies the clinical impact of physiology. Correcting acidemia can lower intracranial pressure in patients with brain injury, even in the presence of hypercapnia.^(6)^ During weaning from mechanical ventilation, normalization of pH may reduce respiratory drive, despite persistent hypercapnia,^(7)^ facilitating ventilator liberation. In *cor-pulmonale*, maintaining normal pH helps prevent pulmonary vasoconstriction and worsening right heart strain.^(8)^

## BARRIERS AND MISSED OPPORTUNITIES

**Figure 1:**
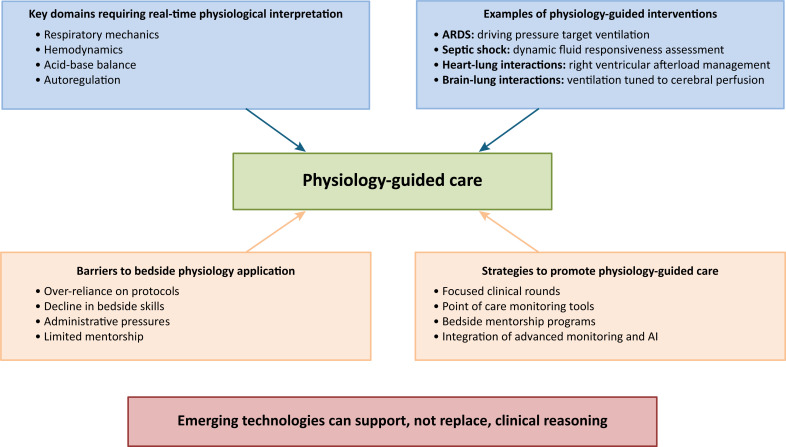
The central role of bedside physiology in critical care.

Even though physiology is considered a cornerstone of medical education, there is concerning evidence that physiological knowledge tends to fade as trainees progress through residency and fellowship. Many critical care fellows struggle to apply fundamental concepts of cardiorespiratory and renal physiology at the bedside, particularly under the pressure of acute decision-making^(9)^ (Figure 1).

AI - Artificial intelligence.

The application of core physiological principles at the bedside enables individualized care, bridging the gap between standardized protocols and patient-specific needs. Mastery of physiology supports informed decision-making in key areas of intensive care, including mechanical ventilation, hemodynamics, acid-base balance, and autoregulation, while overcoming barriers such as over-reliance on protocols, decline in bedside skills, administrative pressures, and lack of mentorship. Integrating physiology-guided care with emerging technologies and fostering structured education will empower young intensivists to improve patient outcomes.

Several systemic and educational factors hinder the practical application of physiology:

– Protocol-driven practice: while protocols help standardize care, they often fail to account for individual patient variability. For instance, applying fixed positive end-expiratory pressure (PEEP) levels without considering chest wall mechanics may be harmful.
– Decline in bedside examination skills: the increasing reliance on advanced imaging and monitoring technologies can displace simple yet essential clinical assessments, such as jugular venous pressure,^(10)^ capillary refill time, and respiratory effort evaluation, which often provide immediate, actionable information.^(5)^
– Administrative pressures: documentation demands, coordination of care, and bureaucratic tasks often consume time and cognitive resources, diverting attention from reflective clinical reasoning and bedside assessment.^(11)^
– Lack of bedside feedback and mentorship: trainees frequently receive feedback on numerical data in conference rooms but lack direct mentorship during physiological assessments. Bedside teaching plays an important role in fostering critical thinking and practical skills.^(12)^

## CLINICAL SCENARIOS HIGHLIGHTING PHYSIOLOGY IN ACTION

### Acute respiratory distress syndrome management

Individualized PEEP titration based on lung mechanics and recruitability improves oxygenation and minimizes ventilator-induced lung injury. Tools such as electrical impedance tomography provide real-time insights into regional lung aeration, guiding safe and effective PEEP adjustments.^(13)^

### Septic shock

After fluid resuscitation and initiation of vasopressors in hypotensive patients, dynamic assessments of fluid responsiveness help prevent unnecessary fluid administration. Persistent hypotension often reflects vasodilation, indicating the need for vasopressors rather than additional fluids.^(14)^

### Heart-lung interactions

Positive pressure ventilation affects venous return and right ventricular afterload. Recognizing these effects - especially in patients with right ventricular dysfunction - through dynamic blood pressure monitoring or echocardiography informs ventilator adjustments and preload management.^(3)^

### Brain-lung interactions

In patients with brain injury and elevated intracranial pressure, fine-tuning ventilation parameters, including PEEP and partial pressure of carbon dioxide (PaCO_2_), based on cerebral perfusion physiology exemplifies the application of integrated, physiology-guided care.^(15)^

## CHALLENGES AND FUTURE DIRECTIONS

Despite its potential, the implementation of physiology-guided care faces several real-world challenges - including monitoring artifacts, patient - ventilator dyssynchrony, and technological limitations in data integration. Educational gaps further hinder consistent application. Moreover, some physiology-based interventions lack validation in large-scale prospective trials, warranting cautious interpretation.

However, emerging technologies, such as advanced monitoring platforms, integrated data systems, and AI-driven analytics, hold promise for helping clinicians interpret complex physiological data. Rather than replacing clinical reasoning, these tools can enhance decision-making and support the application of physiology-based care.

Another promising development is the integration of medical digital twins into intensive care unit care. These computational models dynamically replicate a patient's physiological status using real-time data and historical information, enabling continuous simulation and prediction of responses to interventions. In the context of mechanical ventilation, a digital twin can model lung mechanics, gas exchange, and hemodynamic interactions under various ventilatory settings, assisting clinicians in identifying the safest and most effective strategy tailored to an individual's specific condition. As artificial intelligence, data interoperability, and bedside monitoring advance, the deployment of digital twins could serve as a bridge between evidence-based protocols and precision medicine - potentially reducing complications, optimizing ventilator weaning, and improving long-term outcomes.

## PRACTICAL RECOMMENDATIONS

### For young intensivists

1. Prioritize physiology in both clinical practice and structured learning.
2. Conduct clinical rounds focused on physiology, such as respiratory mechanics, hemodynamics, and acid-base physiology.
3. Use point-of-care tools, including ultrasound, capnography, and ventilator waveform analysis, to gain real-time physiological insights.
4. Establish feedback loops to evaluate the impact of interventions and refine clinical practice.

### For leadership and institutions

1. Promote bedside teaching and mentorship programs focused on applied physiology.
2. Invest in continuing education initiatives that emphasize physiology-guided care.
3. Support the adoption of integrated monitoring systems and decision-support tools.
4. Foster collaborative, multidisciplinary learning environments centered on clinical physiology.
5. Encourage and support research validating physiology-based interventions.

## CONCLUSION

Physiology remains the cornerstone of both the science and art of critical care. In an era marked by rapid technological advances and increasing complexity, the ability to interpret and act on physiological signals will define the next generation of intensivists. By fostering curiosity, promoting mentorship, and honing clinical reasoning, young intensivists can lead the evolution of critical care - grounded in science and driven by bedside practice.

## Data Availability

After publication the data will be available on demand to the authors.
